# BLAT-Based Comparative Analysis for Transposable Elements: BLATCAT

**DOI:** 10.1155/2014/730814

**Published:** 2014-05-18

**Authors:** Sangbum Lee, Sumin Oh, Keunsoo Kang, Kyudong Han

**Affiliations:** ^1^Department of Computer Science, Dankook University, Cheonan 330-714, Republic of Korea; ^2^Department of Nanobiomedical Science and BK21 PLUS NBM Global Research Center for Regenerative Medicine, Dankook University, Cheonan, 330-714, Republic of Korea; ^3^Department of Microbiology, Dankook University, Cheonan 330-714, Republic of Korea; ^4^DKU-Theragen Institute for NGS Analysis (DTiNa), Cheonan 330-714, Republic of Korea

## Abstract

The availability of several whole genome sequences makes comparative analyses possible. In primate genomes, the priority of transposable elements (TEs) is significantly increased because they account for ~45% of the primate genomes, they can regulate the gene expression level, and they are associated with genomic fluidity in their host genomes. Here, we developed the BLAST-like alignment tool (BLAT) based comparative analysis for transposable elements (BLATCAT) program. The BLATCAT program can compare specific regions of six representative primate genome sequences (human, chimpanzee, gorilla, orangutan, gibbon, and rhesus macaque) on the basis of BLAT and simultaneously carry out RepeatMasker and/or Censor functions, which are widely used Windows-based web-server functions to detect TEs. All results can be stored as a HTML file for manual inspection of a specific locus. BLATCAT will be very convenient and efficient for comparative analyses of TEs in various primate genomes.

## 1. Introduction

The advancement of DNA sequencing technology and bioinformatics has tremendously accelerated whole genome sequencing and comparative genomic analysis. Currently, 88 genome sequences are available in the University of California, Santa Cruz (UCSC) Genome Brower website (http://www.genome.ucsc.edu/) [1]. Although the genome database is easily accessible for genome research, data analysis and interpretation still remain challenging due to the amount of sequence data and various research areas within genomics. The UCSC Genome Browser was produced in the early stage of the human genome project and provides optical effects and precise sequence alignments on query sequences [1, 2]. Users can obtain a variety of information including gene tracks, genome conservation, single nucleotide polymorphisms (SNPs), and transposable elements (TEs) from the UCSC Genome Browser [3].

In the human genome, the protein coding regions only account for about 2% of the genome, whereas TEs consist of ~50% of the primate genomes within intragenic and intergenic sequences, which are called noncoding regions [4, 5]. Most studies have focused on the protein coding regions to understand their roles in human health and disease. However, noncoding regions have been emphasized since the ENCyclopedia of DNA Elements (ENCODE) project, which aims to detect new functional sources in the human genomes [6, 7].

To screen TEs in the eukaryote genomes, RepeatMasker (http://www.repeatmasker.org) [8] and Censor (http://www.girinst.org/censor/) [9] web servers have been commonly used. These software tools provide accurate and rapid repetitive DNA annotation results; the UCSC Genome Browser is also connected with them. In the comparative genomic study between six primate whole genome sequences (human, chimpanzee, gorilla, orangutan, gibbon, and rhesus macaque) [10–14], the BLAST-like alignment tool (BLAT) [15] provides an index to find homologous regions from query sequences and allows the manual retrieved alignment of query sequences from the UCSC webpage [3]. However, these processes of manually comparing and retrieving aligned sequences from query sequences are time consuming and difficult to use for novice users.

Here, we propose a handy Windows-based program, BLAT-based comparative analysis for transposable elements (BLATCAT; http://hanlab.dankook.ac.kr/gnu/data/file/Utility/765016963_ExyIiut9_BLATCAT.exe), which automatically and simultaneously performs BLAT, RepeatMasker, and Censor [8, 9, 15]. BLATCAT was developed to detect orthologous regions between the primate genomes. Since other nonprimate species have more genomic diversity and low-quality sequences, it is not accurate to compare with orthologous regions in other nonprimate species. Therefore, BLATCAT compares only six primate genome sequences (human, chimpanzee, gorilla, orangutan, gibbon, and rhesus macaque). These primate genomes are adequate to analyze the evolution of closely related species. The BLATCAT program can significantly reduce serial steps in comparing specific regions of six representative primate genome sequences and support both position and sequence based approach. With these features, the BLACAT program is competitive for comparative analysis of the TE in various primate species.

## 2. Materials and Methods

*Sources*. To obtain comprehensive results, the BLATCAT program utilizes the outputs of the following four popular applications.

### 2.1. UCSC Genome Browser

The UCSC Genome Browser is an interactive website providing useful sequenced-based tools along with a variety of genome sequence data [3]. This website offers useful browsing service for retrieving locations of DNA sequences, gene structures, and distribution of TEs in the genomes by using genomic positions or gene search terms. It currently covers genome sequences of 88 species including the human genome [1].

### 2.2. BLAT Search

BLAT is a pairwise DNA-sequence alignment algorithm that is widely used in comparative genomics [15]. BLAT rapidly identifies similar sequences to a query with high accuracy (>95%). The total limit of multiple query sequences is up to 75,000 letters. BLAT search results display a lot of information as follows: score (calculated according to aligned length and sequence similarity), start (position of first match on the query), end (position of last match on the query), query size (the size of input sequence), identity (sequence similarity), genomic coordinates (genomic positions of the matched sequence), and strand (orientation of the matched sequence in the genome).

### 2.3. RepeatMasker

RepeatMasker [8] is a TE search tool characterizing TEs in given query sequences or genomes. This program uses the Smith-Waterman-Gotoh algorithm, developed by Phil Green (unpublished data). As an input, it accepts both FASTA-formatted sequences and files.

### 2.4. Censor

Censor [9] is also a web-based tool that scans DNA sequences for TEs against a reference dataset of TEs and delivers an abridged annotation of TEs. The major classes of TEs annotated by Censor are 40 subfamilies of DNA transposon and LTR and non-LTR retrotransposons including retroviruses and simple repeats. Censor is also available to screen TEs in other species besides human TEs [16]. It uses the same algorithm with RepeatMasker and supports FASTA, GenBank, and EMBL formats for query sequence.

### 2.5. Development Environment

BLATCAT was developed in the environment as described below (see also Table 1). Since it was implemented in Java (it requires Java Virtual Machine version 1.6 or above) [17], the current executable version of BLATCAT only supports Windows. BLATCAT is implemented with three open libraries called Jsoup, Windowbuilder, and Jsmooth. Briefly, Jsoup (http://jsoup.org) is responsible for interacting with the UCSC genome browser. Windowbuilder (https://www.eclipse.org/windowbuilder) is used to design user interface. An executable version of the BLATCAT program was packed with Jsmooth (http://jsmooth.sourceforge.net).

## 3. Results and Discussion

### 3.1. BLATCAT Workflow

**Figure 1:**
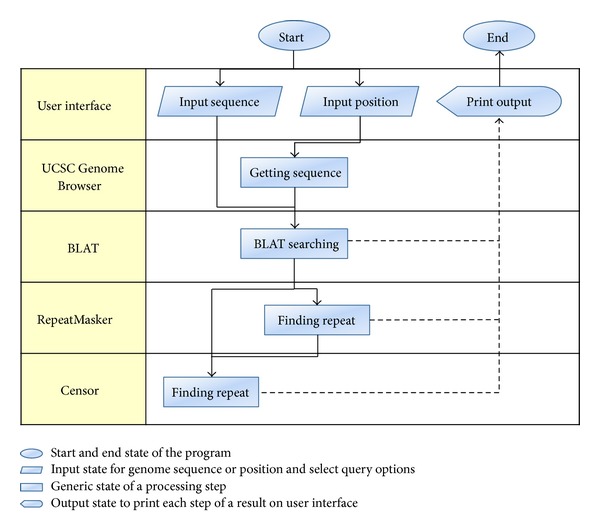
BLATCAT flowchart. BLATCAT runs several programs sequentially and utilizes outputs of the programs. The arrows indicate the flow of the BLATCAT algorithm.

**Figure 2:**
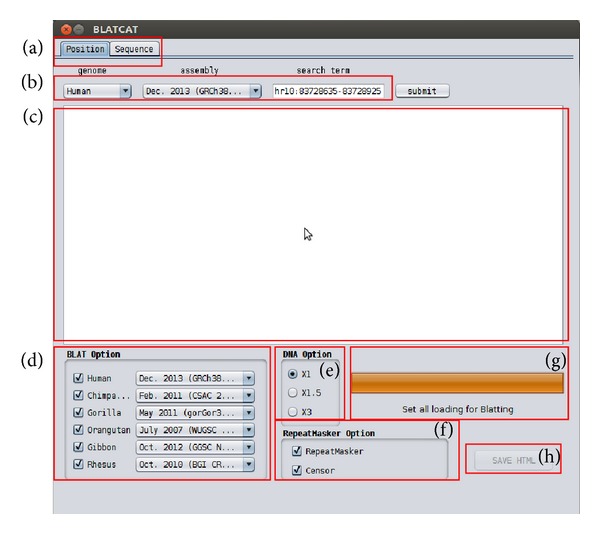
BLATCAT user interface for genomic position. (a) Two types of input tabs are shown. (b) Genome and its assembly version can be changed. Users can put position information in the search term field. (c) Result appears in this field. (d) Selectable species and their genome assembly are shown. (e) The length of a given input sequence can be extended up to three times (×3). Selectable RepeatMasker options (f) and a progress bar (g) are shown. (h) The output can be saved as a HTML file.

**Figure 3:**
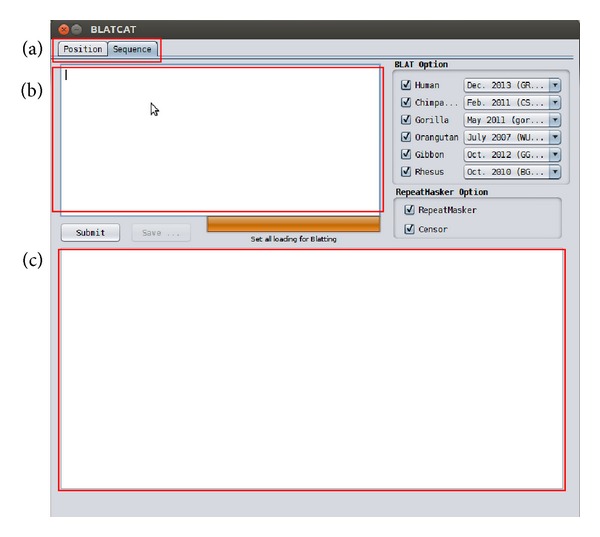
BLATCAT user interface for DNA sequence. (a) DNA sequence can be used as an input for analysis. (b) DNA sequence should be placed in the empty field. (c) Result appears in this empty field.

BLATCAT accepts two types of input: genomic position or DNA sequence (Figures 1 and 2). Users can choose species and different versions of genome assembly for analysis (Figure 2(d)). In addition, the users can extend range of searching regions up to three times by adjusting “DNA option” placed at the bottom (Figure 2(e)). When the user selects the “position” tab for a query with options (Figure 2(a)), BLATCAT first extracts DNA sequences of the given positions (Figure 2(b)) and searches selected genomes for homologous sequences via the UCSC Genome Browser [1]. On the other hand, if the user provides genome sequences instead of the genome positions without any options on the “sequence” tab (Figure 3), the program directly performs pairwise sequence alignment using the BLAT algorithm [15]. Only the most similar sequence is selected and used as a query for searching homologous sequences. Once the homologous sequences are extracted, repetitive DNA sequences in all homologous sequences are identified using RepeatMasker as default [8]. Subsequently, Censor marks TEs in the homologous sequences for visualization [9].

### 3.2. BLATCAT Output

**Figure 4:**
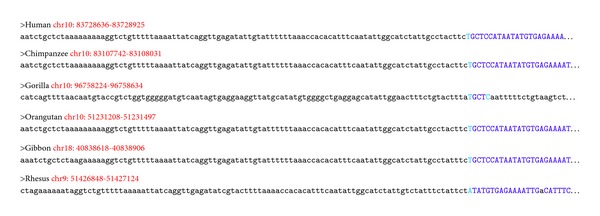
The result of BLAT searching within BLATCAT. Homologous sequence of each species is displayed as FASTA format. Genomic position (red) and repeat sequence (blue) are marked with different colors.

**Figure 5:**
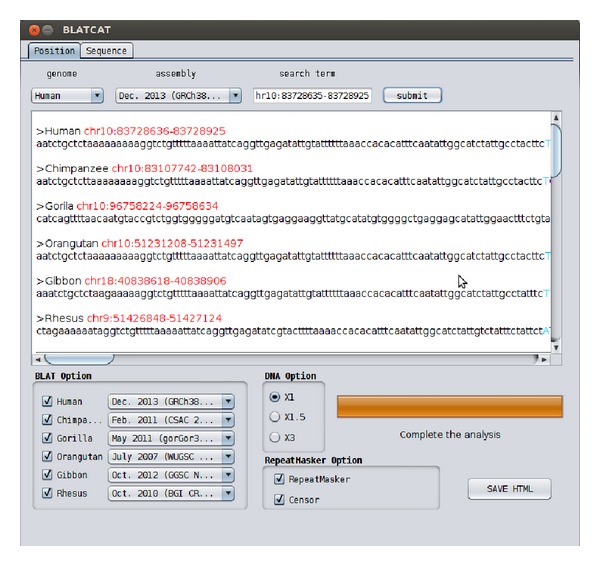
Screenshot of the BLATCAT output. All the results (Figure 4 and Tables 4 and 5, results of BLAT, RepeatMaster, and Censor) are merged and displayed in the user interface at the same time. Other contexts are identical to Figure 2.

The BLATCAT output provides the following useful information for researchers. It shows the homologous sequence and its genomic coordinate in each species (Figure 4). BLATCAT maintains color of strings or formats acquired from other programs, such as the UCSC genome browser, BLAT (Figure 4), RepeatMasker (Table 4), and Censor (Table 5) [1, 8, 9, 15]. These results are merged and displayed at the same time upon submission (Figure 5). Excluding the user interface, all results of previous steps can be stored as a HTML file (Figure 2(h)) if the user clicks the “save HTML” button (Figure 5). Descriptions of attributes of RepeatMasker and Censor can be found in Tables 2 and 3 [8, 9]. The user can easily “copy and paste” any part of the output to other software applications.

### 3.3. Comparison of BLATCAT with the UCSC-BLAT-RepeatMasker-Censor Procedure

Previous studies [18–25] that examined species-specific insertions/deletions mediated by TEs should inspect orthologous primate sequences at each locus using manual methods (UCSC, BLAT, and RepeatMasker/Censor). BLATCAT is a user-friendly program optimized for identifying TEs in homologous sequences of six primate species. The one-step procedure of BLATCAT allows researchers to perform comparative identification of TEs. To obtain TEs in homologous sequences of six species manually, users have to go through several steps. First, the users have to extract DNA sequence of interest from genome browsers, such as UCSC and Ensembl genome (see Figure  S1 in Supplementary Material available online at http://dx.doi.org/10.1155/2014/730814) [1, 26]. Then, homologous sequences are identified by aligning the extracted sequence to the genome of interest by using BLAT or similar programs (Figure  S2). To identify TEs in these sequences, the users have to run RepeatMasker and/or Censor with each homologous sequence as a query repeatedly (Figures  S3 and S4) [8, 9]. These sequential analyses require certain knowledge of algorithms and are time-consuming tasks. Our application explicitly shortens the steps for comparative TE analysis and is easy to use.

To estimate the efficiency of BLATCAT, we compared manual method and BLATCAT in the human position as a query (chr18: 40,208,090–40,208,390). The result indicates that BLATCAT (processing time: 65 sec) works five times faster than that of the manual method (processing time: 356 sec).

### 3.4. The Weaknesses of BLATCAT

Although BLATCAT is a straightforward approach to identify TEs in homolog regions, it also has some weaknesses due to the algorithm. First, BLATCAT requires an Internet connection since it interacts with several web applications. Second, the current version of BLATCAT only runs on the Windows operating system. Third, if the size of input sequence is more than 75,000 bases, it cannot be processed due to the size limitation of the BLAT website. However, most computers are connected to the Internet these days and the typical size of input sequence should be around several kilobases. Fourth, BLATCAT only returns the top-scoring locus of homology found by BLAT, even if there is one or more homologous loci with scores nearly as high as the top hit. Therefore, BLATCAT is comparable to other genomic tools.

## 4. Conclusions

BLAT only finds an orthologous region between a query sequence and another single genome. However, we developed the Windows-based BLATCAT program to simultaneously compare a query sequence with its corresponding sequences from five other primates. In addition, this tool is linked to RepeatMasker and/or Censor to identify full spectrum TEs in the primate genomes. BLATCAT is an easy-to-use tool and is more effective than manual work. Therefore, we believe that BLATCAT is a valuable tool for a comparative analysis of TEs in primate genomes.

## Supplementary Material

Figure S1: Steps to get DNA sequence via the UCSC genome browser.
To obtain the DNA sequence of a given region, the user has to go through the following steps. First, the user should put a genomic coordinate (red box) as a query (top). Second, the user clicks the DNA button (middle). Third, the user selects options and clicks the “get DNA” button (bottom). Then, DNA sequence will appear in FASTA format at a screen.
Figure S2: Retrieve similar DNA sequence using BLAT.
To get similar DNA sequence from other genomes, the following steps need to be done through BLAT. First, the user should select the BLAT button in the UCSC genome browser and paste the DNA sequence into the empty box with the appropriate options (top). Second, the user has to click the details link in the BLAT search results after the completion of BLAT searching (middle). Finally, the user can get the similar sequence to the query sequence from the result (bottom). The identical parts of this sequence will be marked in blue.
Figure S3: TE identification using Repeat Masker.
To identify TEs in the ortholog sequence, the user has to obtain the ortholog sequence from BLAT as the FASTA format. Then the ortholog sequence can be used as a query sequence (top). After the completion of analysis, result will be appeared at a screen (bottom). To obtain TEs from different genomes, the user has to repeat this analysis manually.
Figure S4: TE identification using Censor.
To identify TEs in the ortholog sequence using Censor, the user has to follow similar steps as mentioned in Figure S3.

## Figures and Tables

**Table 1:** List of developmental libraries implemented in BLATCAT.

Development tool	Eclipse Indigo version Java EE IDE
Development language	Java (JDK 1.6)
Used library	Jsoup, Windowbuilder, and Jsmooth

**Table 2:** Description of the RepeatMasker attributes.

Attribute	Description
SW score	Smith-Waterman score of the match, usually complexity adjusted
Perc div.	Percentage of substitutions in matching region compared to the consensus
Perc del.	Percentage of bases opposite a gap in the query sequence (deleted bp)
Perc ins.	Percentage of bases opposite a gap in the repeat sequence (inserted bp)
Query sequence	Name of query sequence
Position in query	
Begin	Starting position of match in query sequence
End	Ending position of match in query sequence
(Left)	Number of bases in query sequence past the ending position of match
Matching repeat	Match is with the complement of the consensus sequence in the database
Repeat class/family	Name of the matching interspersed repeat
Position in repeat	
Begin	The class of the repeat
End	Number of bases in (complement of) the repeat consensus sequence prior to beginning of the match
(Left)	Starting position of match in database sequence (using top-strand numbering)
ID	Ending position of match in database sequence

**Table 3:** Description of the Censor attributes.

Attribute	Description
Name	Column Name contains locus names of submitted query sequences (first column) and Repbase library sequences (fourth column). Repbase names are hyperlinked to their sequences.
From/To	Column From/To contains beginning/ending of positions of fragment on corresponding sequence.
Class	This is class/subclass of repeat as specified in repeat annotation.
Dir	Values in column Dir indicate orientation (“d” for direct and “c” for complementary) of repeat fragment—columns 4–6.
Sim	Column Sim contains value of similarity between 2 aligned fragments.
Pos	Column Pos is roughly the ratio of positives to alignment length.
Mn : Ts	Column Mm : Ts is a ratio of mismatches to transitions in nucleotide alignment. The closer this number is to 1 the more likely is that mutations are evolutionary.
Score	This column contains the alignment score obtained from blast.

**Table 4:** The result of RepeatMasker within BLATCAT.

SW	perc	perc	perc	query	position in query				Matching repeat		Position in repeat			ID
Score	Div.	Del.	Ins.	Sequence	Begin	End	(Left)		Repeat	Class/family	Begin	End	(Left)	
510	28.2	6.4	4.5	Human	10	355	(135)	C	HAL1b	LINE/L1	(406)	2015	1664	5
475	28.7	6.4	4.2	Chimpanzee	10	355	(135)	C	HAL1b	LINE/L1	(405)	2016	1664	1
792	20.5	1.3	0.0	Gorilla	1	151	(460)	+	L1MC1	LINE/L1	6176	6328	(5)	3
402	29.3	7.3	4.8	Gorilla	133	476	(135)	C	HAL1b	LINE/L1	(406)	2015	1664	4*
478	28.6	6.7	4.8	Orangutan	10	355	(135)	C	HAL1b	LINE/L1	(406)	2015	1664	6
465	29.1	6.6	4.3	Gibbon	11	373	(116)	C	HAL1b	LINE/L1	(406)	2015	1645	2
319	32.7	6.6	2.1	Rhesus	24	342	(135)	C	HAL1b	LINE/L1	(425)	1996	1664	7

The RepeatMasker output is displayed. Descriptions of the attributes can be found in Table 1.

*indicates that there is a higher-scoring match whose domain partly (<80%) includes the domain of this match [8].

**Table 5:** The result of Censor within BLATCAT.

Name	From	To	Name	From	To	Class	Dir	Sim	Pos/Mm : Ts	Score
Human (SVG plot; alignments; masked)										
Human	10	368	HAL1B	610	973	NonLTR/L1	c	0.7003	2.0667	774

Chimpanzee (SVG plot; alignments; masked)										
Chimpanzee	10	368	HAL1B	610	974	NonLTR/L1	c	0.6955	2.0652	745
Chimpanzee	386	434	Gypsy-2_HMM-I	5194	5247	LTR/Gypsy	c	0.8039	1.6	209

Gorilla (SVG plot; alignments; masked)										
Gorilla	1	151	L1MC1	923	1075	NonLTR/L1	d	0.7843	1.3478	757
Gorilla	154	489	HAL1B	610	953	NonLTR/L1	c	0.6907	1.8936	674

Orangutan (SVG plot; alignments; masked)										
Orangutan	10	361	HAL1B	617	973	NonLTR/L1	c	0.7064	1.8298	761
Orangutan	386	434	Gypsy-2_HMM-I	5194	5247	LTR/Gypsy	c	0.8039	1.6	209

Gibbon (SVG plot; alignments; masked)										
Gibbon	11	367	HAL1B	610	973	NonLTR/L1	c	0.6966	1.9375	765
Gibbon	385	433	Gypsy-2_HMM-I	5194	5247	LTR/Gypsy	c	0.8039	1.6	209

Rhesus (SVG plot; alignments; masked)										
Rhesus	24	355	HAL1B	610	954	NonLTR/L1	c	0.6677	1.9231	606

The Censor output is shown. Each table shows the result of each species obtained from the Censor analysis.
