# Supplementary material for: BLAT-Based Comparative Analysis for Transposable Elements: BLATCAT
Source: Biomed Res Int. 2014 May 18;2014:730814. doi: 10.1155/2014/730814 (PMC4052159; doi:10.1155/2014/730814)
Supplement: Supplementary file 1 — Figure S1: Steps to get DNA sequence via the UCSC genome browser. To obtain the DNA sequence of a given region, the user has to go through the following steps. First, the user should put a genomic coordinate (red box) as a query (top). Second, the user clicks the DNA button (middle). Third, the user selects options and clicks the “get DNA” button (bottom). Then, DNA sequence will appear in FASTA format at a screen. Figure S2: Retrieve similar DNA sequence using BLAT. To get similar DNA sequence from other genomes, the following steps need to be done through BLAT. First, the user should select the BLAT button in the UCSC genome browser and paste the DNA sequence into the empty box with the appropriate options (top). Second, the user has to click the details link in the BLAT search results after the completion of BLAT searching (middle). Finally, the user can get the similar sequence to the query sequence from the result (bottom). The identical parts of this sequence will be marked in blue. Figure S3: TE identification using Repeat Masker. To identify TEs in the ortholog sequence, the user has to obtain the ortholog sequence from BLAT as the FASTA format. Then the ortholog sequence can be used as a query sequence (top). After the completion of analysis, result will be appeared at a screen (bottom). To obtain TEs from different genomes, the user has to repeat this analysis manually. Figure S4: TE identification using Censor. To identify TEs in the ortholog sequence using Censor, the user has to follow similar steps as mentioned in Figure S3. [file 730814.f1.pdf]

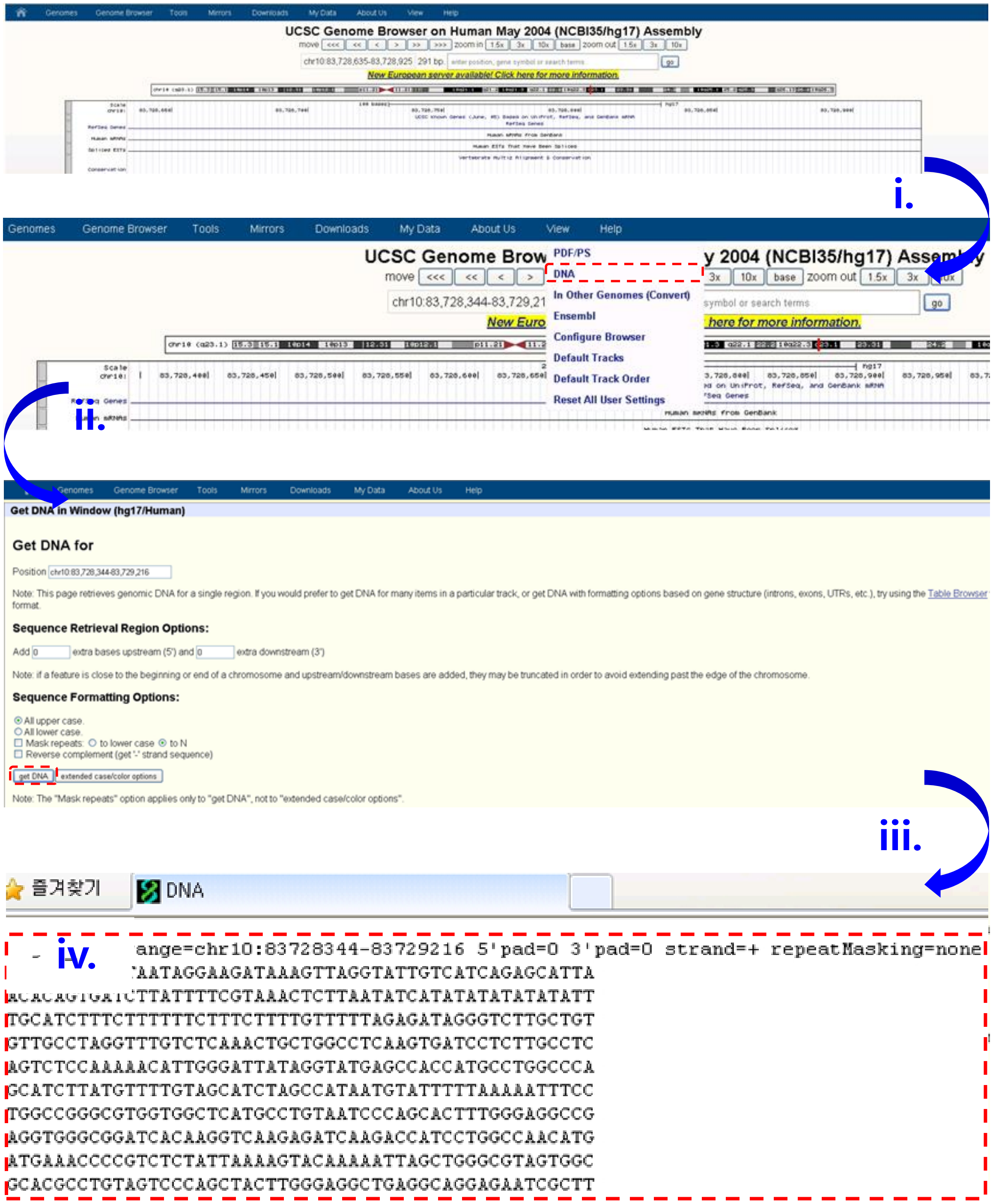

Figure S1. Steps to get DNA sequence via the UCSC genome browser.

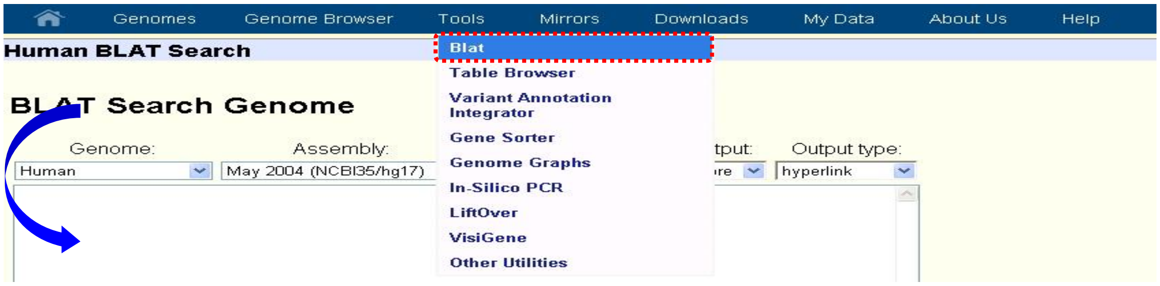

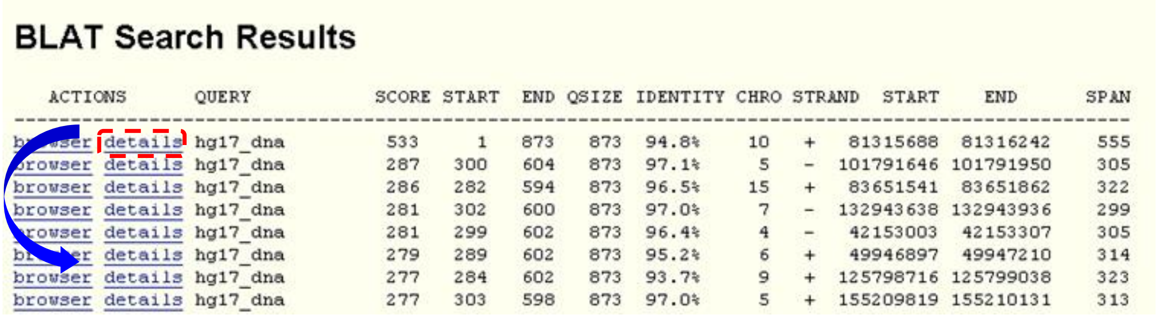

BLAT Search Results

ACTIONS		QUERY	SCORE	START	END	QSIZE	IDENTITY	CHRO	STRAND	START	END	SPAN
browser	details	hg17_dna	533	1	873	873	94.8%	10	+	81315688	81316242	555
browser	details	hg17_dna	287	300	604	873	97.1%	5	-	101791646	101791950	305
browser	details	hg17_dna	286	282	594	873	96.5%	15	+	83651541	83651862	322
browser	details	hg17_dna	281	302	600	873	97.0%	7	-	132943638	132943936	299
browser	details	hg17_dna	281	299	602	873	96.4%	4	-	42153003	42153307	305
browser	details	hg17_dna	279	289	602	873	95.2%	6	+	49946897	49947210	314
browser	details	hg17_dna	277	284	602	873	93.7%	9	+	125798716	125799038	323
browser	details	hg17_dna	277	303	598	873	97.0%	5	+	155209819	155210131	313

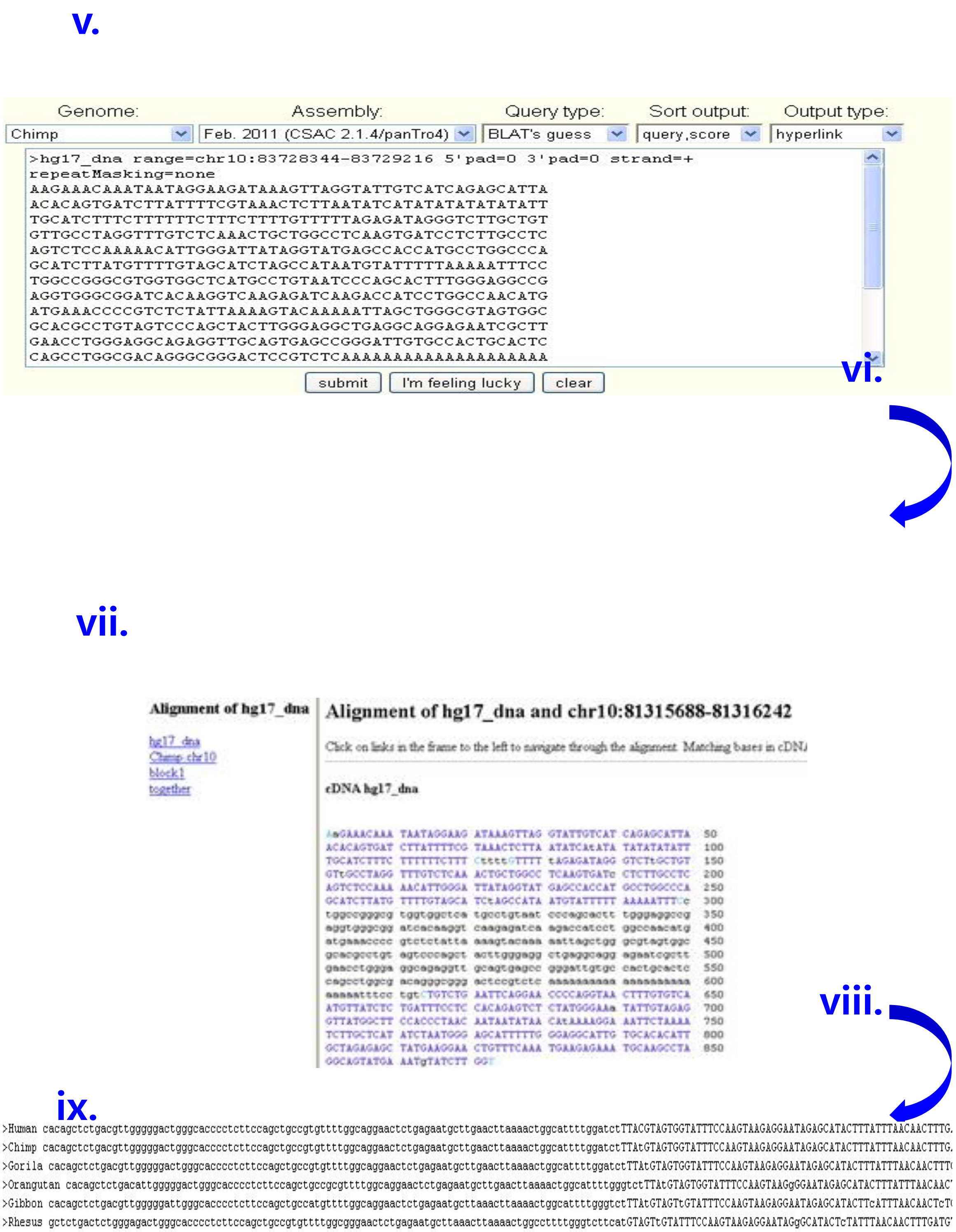

Figure S2. Retrieve similar DNA sequence using BLAT.

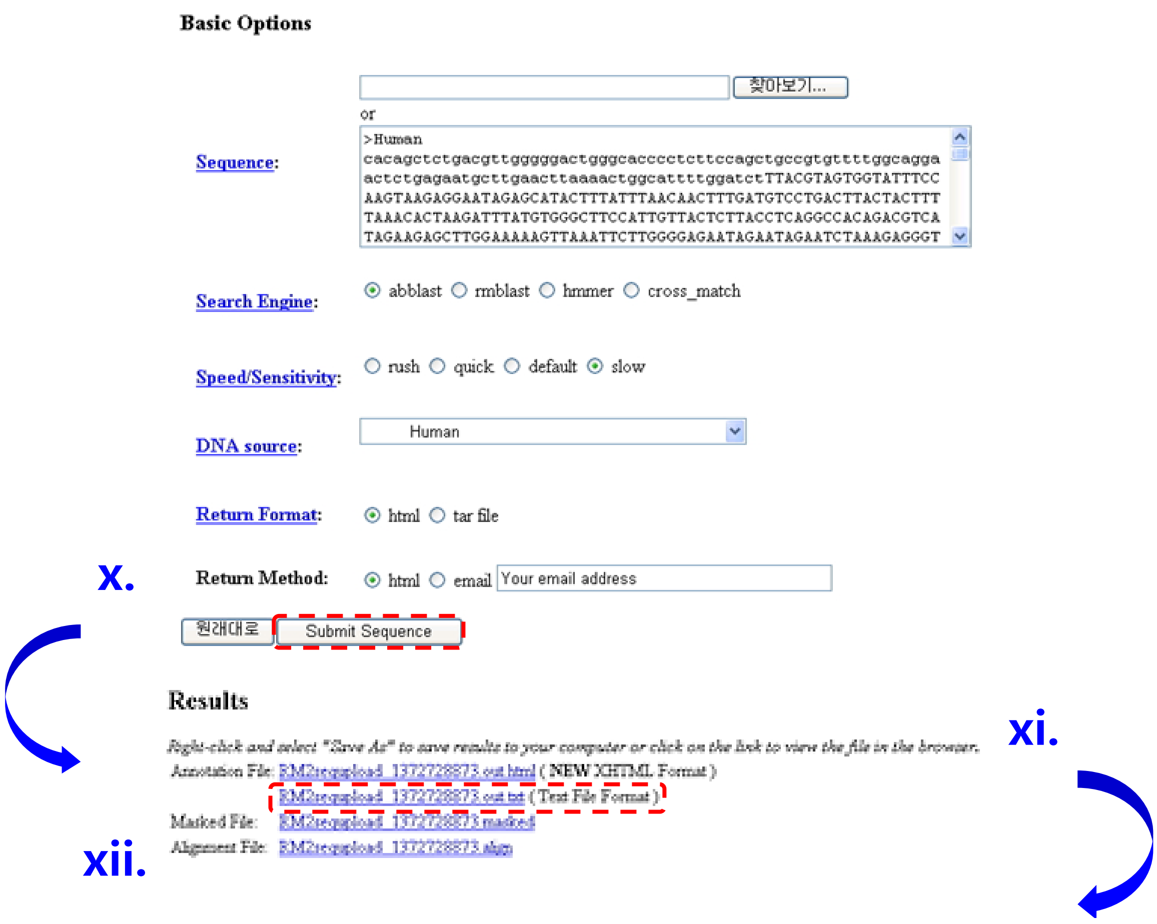

SW score	perc div.	perc del.	perc ins.	query sequence	position in query begin	end	(left)		matching repeat	repeat class/family	position in repeat begin	end	(left)	ID
551	26.1	8.1	3.2	Chimp	17	226	(844)	+	MER115	DNA/hAT-Tip100	450	669	(24)	1
17	18.5	0.0	0.0	Chimp	802	832	(238)	+	A-rich	Low_complexity	1	31	(0)	2
17	12.1	2.4	7.0	Chimp	959	1000	(70)	+	GA-rich	Low_complexity	1	43	(0)	3
575	26.1	9.7	1.3	Gibbon	32	248	(821)	+	MER115	DNA/hAT-Tip100	459	693	(0)	4
17	18.5	0.0	0.0	Gibbon	801	831	(238)	+	A-rich	Low_complexity	1	31	(0)	5
17	12.1	2.4	7.0	Gibbon	958	999	(70)	+	GA-rich	Low_complexity	1	43	(0)	6
564	27.5	8.2	2.9	Gorila	17	248	(822)	+	MER115	DNA/hAT-Tip100	450	693	(0)	7
17	18.5	0.0	0.0	Gorila	802	832	(238)	+	A-rich	Low_complexity	1	31	(0)	8
15	12.8	2.5	7.3	Gorila	959	998	(72)	+	GA-rich	Low_complexity	1	41	(0)	9
542	28.4	8.2	2.9	Human	17	248	(822)	+	MER115	DNA/hAT-Tip100	450	693	(0)	10
17	18.5	0.0	0.0	Human	802	832	(238)	+	A-rich	Low_complexity	1	31	(0)	11
15	12.8	2.5	7.3	Human	959	998	(72)	+	GA-rich	Low_complexity	1	41	(0)	12
597	27.8	7.8	2.5	Orangutan	17	248	(814)	+	MER115	DNA/hAT-Tip100	450	693	(0)	13
17	14.6	2.3	6.7	Orangutan	951	994	(68)	+	GA-rich	Low_complexity	1	45	(0)	14
609	24.2	10.2	1.4	Rhesus	28	232	(898)	+	MER115	DNA/hAT-Tip100	459	681	(12)	15

Figure S3. TE identification using RepeatMasker.

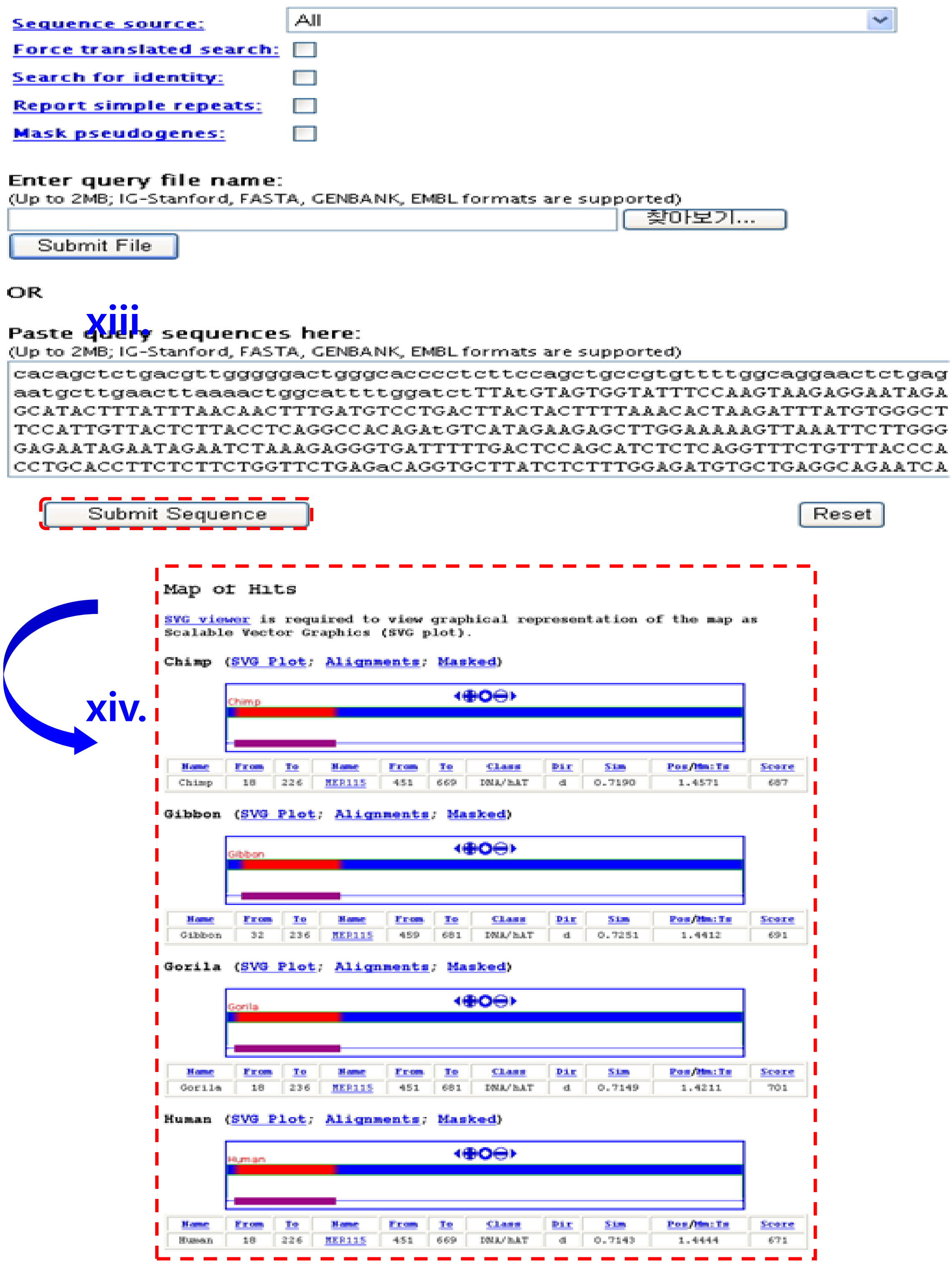

Figure S4. TE identification using Censor
